# Genome Assembly and Genetic Traits of the Pleuromutilin-Producer *Clitopilus passeckerianus* DSM1602

**DOI:** 10.3390/jof8080862

**Published:** 2022-08-16

**Authors:** Thomas Schafhauser, Daniel Wibberg, Antonia Binder, Christian Rückert, Tobias Busche, Wolfgang Wohlleben, Jörn Kalinowski

**Affiliations:** 1 Mikrobiologie und Biotechnologie, Interfakultäres Institut für Mikrobiologie und Infektionsmedizin, Eberhard Karls Universität Tübingen, Auf der Morgenstelle 28, 72076 Tuebingen, Germany; 2 Centrum für Biotechnologie, CeBiTec, Universität Bielefeld, Universitätsstr. 27, 33615 Bielefeld, Germany; 3 Institute of Bio- and Geosciences IBG-5, Computational Metagenomics, Forschungszentrum Jülich GmbH, 52425 Juelich, Germany; 4 Institut für Mikrobiologie, Technische Universität Dresden, Zellescher Weg 20b, 01062 Dresden, Germany; 5 Cluster of Excellence EXC 2124—Controlling Microbes to Fight Infections, 72076 Tuebingen, Germany; 6 German Center for Infection Research (DZIF), Partner Site Tübingen, 72076 Tuebingen, Germany

**Keywords:** genome assembly, Nanopore sequencing, Illumina sequencing, *Clitopilus*, Agaricales, mitochondrial genome, heteroplasmic mycelium, pleuromutilin, terpene synthase, biosynthetic gene cluster

## Abstract

The gilled mushroom *Clitopilus passeckerianus* (Entolomataceae, Agaricales, Basidiomycota) is well known to produce the terpenoid pleuromutilin, which is the biotechnological basis for medically important antibiotics such as lefamulin and retapamulin. Their unique mode of action and good tolerance entails an increasing demand of pleuromutilin-derived antibiotics in veterinary and human health care. Surprisingly, despite their pharmaceutical importance, no genome sequence is available of any pleuromutilin-producing fungus. Here, we present the high-quality draft genome sequence of the pleuromutilin-producer *C. passeckerianus* DSM1602 including functional genome annotation. More precisely, we employed a hybrid assembly strategy combining Illumina sequencing and Nanopore sequencing to assemble the mitochondrial genome as well as the nuclear genome. In accordance with the dikaryotic state of the fungus, the nuclear genome has a diploid character. Interestingly, the mitochondrial genome appears duplicated. Bioinformatic analysis revealed a versatile secondary metabolism with an emphasis on terpenoid biosynthetic enzymes in *C. passeckerianus* and also in related strains. Two alleles of biosynthetic gene clusters for pleuromutilin were found in the genome of *C. passeckerianus*. The pleuromutilin genes were reassembled with yeast-specific elements for heterologous expression in *Saccharomyces cerevisiae*. Our work lays the foundation for metabolic strain engineering towards higher yields of the valuable compound pleuromutilin.

## 1. Introduction

The basidiomycete *Clitopilus passeckerianus* is a saprotrophic fungus belonging to the family of Entolomataceae within the fungal order Agaricales, also known as gilled mushrooms. *Clitopilus* species are especially endemic in northern temperate areas [1]. *C. passeckerianus* has been named after its discoverer Friedrich Passecker, an Austrian agronomist, who encountered it as a contamination in agricultural mushroom farms [2]. The fungus occasionally also grows on wood and old horse dung [3], and has even been reported from deep mining galleries [2].

Morphologic and genetic observations [2,4,5,6,7] reveal that *C. passeckerianus* undergoes the typical life cycle of Agaricales. Its main form of appearance is the dikaryotic mycelium composed of microscopic hyphae harboring two distinct haploid nuclei [4,5,6,7]. The dikaryon is able to differentiate into macroscopic fruiting bodies [2,7], which are characterized by a tiny stipe, a small white cap, and amber-to-pinkish gills [2]. The gill tissue generates slightly pink-toned [2], haploid basidiospores upon meiosis [7]. The spores germinate on a suitable substrate and produce a haploid mycelium—the monokaryon [7]. When two compatible monokaryons meet and fuse, the life cycle is completed as the dikaryotic mycelium—populated by haploid nuclei of both parental genotypes—is rebuilt [7]. As eukaryotes, fungal cells contain a mitogenome besides the nuclear genome. The mitochondrial DNA (mtDNA) in Agaricales is usually inherited uniparentally (i.e., from one of the parental monokaryons) to give rise to a homoplasmic dikaryon containing only a single type of mtDNA throughout the whole mycelium [8,9,10]. The mtDNA and its inheritance in *C. passeckerianus* has not yet been investigated.

*Clitopilus passeckerianus* are famous for the production of pleuromutilin, a tricyclic diterpene antibiotic of great medicinal importance, which interferes with protein synthesis of mainly Gram-positive bacteria [11]. Pleuromutilin was first isolated 70 years ago in the golden age of antibiotic discovery from *C. passeckerianus* and *C. scyphoides* [11]—at that time both assigned to the genus *Pleurotus*, hence the compound’s name, pleuromutilin. Since then, several pleuromutilin-producing strains have become known, which form a tight monophyletic clade within the Entolomataceae, suggesting that pleuromutilin production has only evolved once [5]. Although it has been used in veterinary medicine for decades, pleuromutilin has just recently been applied in human medicine in the form of the semi-synthetic derivatives retapamulin (for topic application) and lefamulin (for systemic application). Despite the longtime usage in livestock, resistance against pleuromutilin is very rare and develops rather slowly [12,13]. Moreover, the unique binding site of pleuromutilins at the 23 S rRNA of bacterial ribosome 50 S subunit drastically reduces the chance of cross-resistance to other antibiotics [14,15]. These favorable features together with their good tolerance make pleuromutilins precious drugs in human health care.

The biosynthetic pathway of pleuromutilin has been elucidated simultaneously in *C. passeckerianus* and the related strain *C. pseudo-pinsitus* by two research groups [4,16,17] via the consecutive expression of the involved genes in *Aspergillus oryzae*. According to that, seven *ple* genes encode (i) a geranyl-geranyl diphosphate (GGPP) synthase responsible for combining four C-5 isoprene units to the linear C-20 GGPP, (ii) a terpene synthase catalyzing the key reaction and committed step from GGPP to the tricyclic compound premutilin, and (iii) finally tailoring enzymes such as P450 monooxygenases, a dehydrogenase, and an acetyl-transferase altogether generating the final product pleuromutilin. In both the genomes of *C. passeckerianus* and *C. pseudo-pinsitus*, the *ple* genes are organized in a biosynthetic gene cluster (BGC) with identical architecture [4,17].

The fungus *C. passeckerianus* is genetically tractable using polyethylene glycol-mediated or *Agrobacterium*-mediated transformation [6]. Moreover, molecular tools for gene manipulation such as dominant selection markers and a gene silencing system have been developed [6]. However, all attempts aimed at increasing pleuromutilin production—e.g., by inserting additional biosynthetic genes—were unsuccessful, supposedly due to problems related to the nuclear redundancy [7] and a phenomenon called sense suppression [4]. In contrast, heterologous expression of the pleuromutilin BGC in the ascomycete host *Aspergillus oryzae* led to a stable and increased pleuromutilin production [4,17]. These studies constitute the first gene clusters from a basidiomycete to be successfully expressed in an ascomycete. Besides *A. oryzae*, no other host strain has been tested for heterologous pleuromutilin production.

The availability of complete fungal genomes is expanding rapidly; however, basidiomycetes still are represented only moderately—the Joint Genome Institute [18] currently lists 624 basidiomycete genomes, among them 183 from Agaricales, in contrast to 1448 ascomycete genomes [19]. Surprisingly, despite the pharmaceutical importance of pleuromutilin-producing species, no genome sequence is available from any such fungi. Indeed, the root endophyte and non-producer *C. hobsonii* is the only member of the family Entolomataceae whose genome has been sequenced. To facilitate genome assembly though, the DNA has been extracted from the haploid monokaryon instead of the dikaryotic mycelium of *C. hobsonii* [20].

In this study, we established high-quality nuclear and mitochondrial genome sequences of the dikaryotic fungus *C. passeckerianus* DSM1602 including functional genome annotation. We performed phylogenetic and comparative genome analysis with related gilled mushrooms to estimate genomic similarity of the analyzed fungi. Furthermore, our study provides insights into a pronounced secondary metabolism of *C. passeckerianus* and related strains with an emphasis on terpenoid BGCs. The high-quality *C. passeckerianus* genome will help to investigate fundamental traits in Agaricales genetics and it paves the way for biotechnological approaches that aim at metabolic pathway engineering or complete BGC refactoring of enzymatic pathways such as the pleuromutilin biosynthesis.

## 2. Materials and Methods

### 2.1. Clitopilus passeckerianus Growth and DNA Isolation

The strain *C. passeckerianus* DSM1602 (=ATCC, NRRL 3100), obtained from the German Collection of Microorganisms and Cell Cultures (DSMZ), was routinely grown in 50 mL potato dextrose broth (PDB; Karl Roth GmbH, Karlsruhe, Germany) in a 100 mL baffled flask at 180 rpm and 25 °C. Whole genome DNA was isolated and purified with the ‘NucleoBond HMW DNA’ kit from Macherey-Nagel (Düren, Germany) according to the manufacturer’s instructions. The isolated DNA was used for sequencing when the required spectroscopic criteria were met (260/280 ratio value: ~1.8; 260/230 ration value: 2.0–2.2) and when a 0.5% agarose control gel confirmed the presence of large DNA fragments (>>10 kbp) and the absence of visible amounts of RNA.

### 2.2. Intron/Exon Analysis of Ple Genes in C. passeckerianus

For intron/exon analysis of the pleuromutilin biosynthesis genes *ple*, RNA was isolated from cultures grown for approx. 7 days in PDB using the ‘Quick-RNA Fungal/Bacterial Kit’ (cat no R2014; Zymo Research, Freiburg, Germany). Isolated RNA was treated with DNase (cat no AM1906, Thermo Fisher Scientific) and reverse transcribed to cDNA using the ‘RevertAid RT Kit’ (cat no K1691, Thermo Fisher Scientific, Waltham, MA, USA) with anchored oligo(dT)18-primers. Gene transcripts were PCR-amplified with the Q5 HF DNA polymerase (cat no M0491, New England Biolabs, Ipswich, MA, USA) using primers listed in Tables S1 and S2, sub-cloned using the ‘CloneJet PCR Cloning Kit’ (cat no K1231, Thermo Fisher Scientific), and Sanger-sequenced at Eurofins Genomics (Ebersberg, Germany).

### 2.3. Nanopore Library Preparation and GridION Sequencing

A sequencing library with genomic DNA of *C. passeckerianus* DSM1602 was prepared as recently described for other species [21,22]. In brief, the Nanopore DNA Sequencing kit (SQK-LSK09, Oxford Nanopore Technologies [ONT], Oxford, UK) was used according to the manufacturer’s instructions. Sequencing was performed on an ONT GridION Mk1 sequencer using an R9.4.1 flow cell according to the manufacturer’s instructions.

### 2.4. Illumina Library Preparation and MiSeq Sequencing

Whole-genome shotgun PCR-free libraries of *C. passeckerianus* DSM1602 for Illumina sequencing were constructed as described before for other species [22]. Quality of the resulting libraries was controlled by using an Agilent 2000 Bioanalyzer with an Agilent High Sensitivity DNA Kit (Agilent Technologies, Santa Clara, CA, USA) for fragment sizes of 500—1000 bp. Paired-end sequencing was performed on the Illumina MiSeq platform (2 × 300 bp, v3 chemistry). Adapters and low-quality reads were removed by an in-house software pipeline prior to polishing, as recently described [23].

### 2.5. Base Calling, Reads Processing, and Assembly

The software MinKNOW 3.6.5. (ONT) was used to control the run using the 48 h sequencing run protocol. Base calling was performed offline using guppy 3.2.10. The assembly was performed using Canu v2.1.1 [24]. The resulting contigs were polished with the Illumina reads using Pilon [25], which was run for ten iterative cycles. BWA-MEM [26] was used for read mapping in the first five iterations and Bowtie2 v2.3.2 [27] was used in the remaining five iterations.

### 2.6. Gene Prediction and Genome Annotation

Gene prediction was performed by applying GeneMark-ES (v4.6.2) [28] using default settings and the fungi mode. Predicted genes were functionally annotated using a modified version of the genome annotation platform GenDB 2.0 [29] for eukaryotic genomes as previously described [30]. For automatic annotation within the platform, similarity searches against different databases, including COG [31], KEGG [32], and SWISS-PROT [33], were performed. In addition to genes, putative tRNA genes were identified with tRNAscan-SE [34]. Completeness, contamination, and strain heterogeneity were estimated with BUSCO (v3.0.2) [35], using the fungi-specific single-copy marker genes database (odb9).

### 2.7. Comparative Genome Analyses and Phylogenetic Analysis

Genome comparisons and investigations of phylogeny were accomplished using a modified version of the comparative genomics program EDGAR designed to handle eukaryotic genomes and their multi-exon genes [36], as described recently [21,22]. Average nucleotide identity analysis (ANI), average amino acid identity analysis (AAI), and percentage of conserved proteins (POCP) analysis were performed, as described previously [21,22,37,38].

### 2.8. Identification and Analysis of the Mitogenome

The mitogenome was identified on the basis of two particular criteria. In basidiomycetes, the mitochondrial DNA (mtDNA) generally features a lower GC content than the nuclear genome [39]. Therefore, on the one hand, contigs were identified whose GC content clearly deviated from the average GC content. On the other hand, contigs that were over-represented were considered to belong to the mtDNA since eukaryotes usually contain multiple mitochondria per cell [40]. This led to a single contig that fulfilled both criteria. This contig represents the mitogenome. Subsequently, automatic gene prediction and annotation were performed using the genome annotation systems GenDB 2.0 [29] and Prokka [41]. Intergenic regions were analyzed by BLAST programs [42] for any additional coding sequences that might have been missed in preceding analyses. Sequence interpretation was refined manually by means of splice-site analyses, as described before [43].

### 2.9. Construction of the Yeast-PleBGCs

The yeast-pleBGCs consist of 16–22 discrete DNA elements that were PCR-amplified (Q5 DNA polymerase, New England Biolabs, Ipswich, MA, USA) using either cDNA of *C. passeckerianus* or DNA molecules synthesized by Biocat (Heidelberg, Germany) as a template. The DNA fragments were then step-wise assembled in vitro (see Figure S2 for details) and propagated in *Escherichia coli* ‘NovaBlue Singles Cells Novagen’ (Merck, Darmstadt, Germany). First, in the case of yeast-pleBGC-C, the fragments were fused pairwise by fusion PCR (overlap extension PCR, see Table S3). Next, between three and five DNA fragments were assembled by Gibson assembly (NEBuilder HiFi DNA assembly mix, cat no E2621, New England Biolabs) and introduced at the same time in the vector pBluescript, which was linearized with EcoRV. Finally, the assembled fragments were excised from pBluescript using type IIS restriction enzymes to generate DNA fragments (four per assembly) without remnants of the restriction site. This allowed seamless Gibson assembly of the excised large fragments with an EcoRI/Kpn2I-fragment of pBR322. The EcoRI/Kpn2I-fragment of pBR322 contains an ampicillin resistance cassette and an origin of replication for selection and propagation in *E. coli* (see Figure S3). The sequences of the yeast-pleBGCs are available as genbank-files in the Supplementary Materials.

## 3. Results and Discussion

### 3.1. Generation of a High-Qualitiy Draft Genome of Clitopilus passeckerianus DSM1602

In order to decipher the complete genome sequence of *Clitopilus passeckerianus* DSM1602, third-generation Nanopore sequencing together with second-generation Illumina sequencing were applied. Nanopore sequencing resulted in 22.81 Gb, which were assembled into 166 high-quality contigs with a total size of 65.6 Mb and an average GC content of 49.4%. For the final assembly, the contigs obtained from Nanopore sequencing were polished with raw data from a whole-genome shotgun Illumina paired-end sequencing approach. In total, 27,568 SNPs, 30 ambiguous bases, 88,514 small insertions, and 8213 small deletions were identified and corrected by this polishing approach. The combination of long-read (Nanopore) and short-read (Illumina) sequencing improved base accuracy and significantly reduced error rates in the final genome.

The obtained genome sequence was applied to automatic annotation as described in the Section 2 which resulted in 23,566 protein coding sequences (CDS), 422 tRNA genes, and 126 rRNA genes (Table 1).

A BUSCO analysis was performed to estimate the genomic data quality. This analysis revealed that 93.4% (284 of 303) of eukaryotic core genes—defined by the BUSCO dataset [35]—were completely detected, 2.0% partially, and only 4.0% were missing. According to our experience, fungal genomes can be regarded as fully covered when the value is above 90%. Therefore, the assembled genome sequence of *C. passeckerianus* is assumed to be complete.

In summary, the combination of Illumina and Nanopore sequencing resulted in a high-quality and state-of-the-art fungal draft genome sequence highly suitable for further genome analysis.

### 3.2. The C. passeckerianus Genome Has a Diploid Character

The DNA used for sequencing was isolated from untreated dikaryotic mycelium that contained a set of two distinct haploid nuclei. Therefore, genes were expected to be present in allelic variants and the sequence dataset should have had a diploid character. To verify this, several analyses were performed. In a first analysis, all contigs were compared with each other by BLAST in order to detect local similarities. It was observed that a large number of contigs share similarity with (at least) one other contig at regions of more than 1000 bp of lengths. The largest matching region is longer than 100 kb, featuring a sequence identity of 99%. The two corresponding contigs likely display (parts of) homologous chromosomes of the two haploid nuclei. Apparently, this contig pair contains allelic variants of genes that are significantly different to each other, which is why they were not assembled into one contig. Moreover, the occurrence of sequence differences is not evenly distributed over the aligned contig pair, indicating that some regions are more conserved than others.

In a second analysis, the BUSCO results (see above) were investigated for duplications. It turned out that among the identified eukaryotic core genes approx. 50% appeared twice—which strongly points to the presence of homologous chromosomes. The reason that the duplication of core genes is not closer to 100% can be explained by the fact that the other half of the core genes is obviously very highly conserved so that no allelic variants can be discriminated and hence they appear only once in the screening.

**Figure 1:**
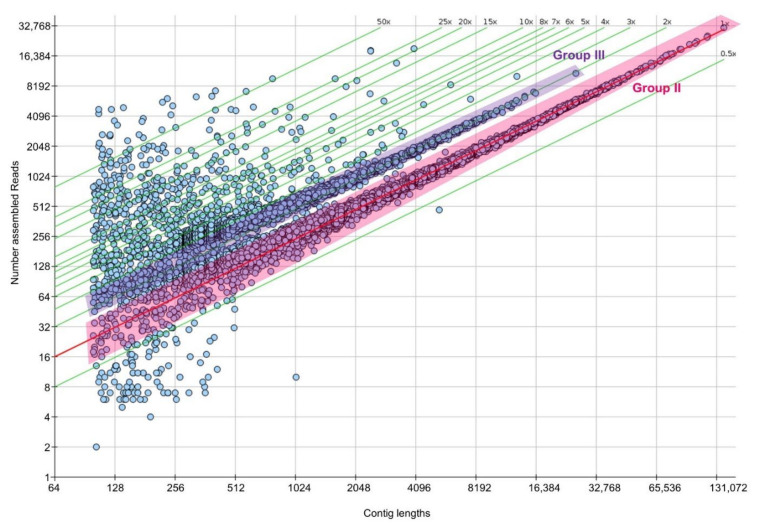
Contig-length vs. read-count plot. Dots represent the length of a contig (*x*-axis, in bp) plotted against the number of reads (*y*-axis) assembled into that contig. The red line displays the calculated coverage of unique contigs (1×), green lines display multiples of that coverage rate. Several contig groups can be discriminated: (I) underrepresented contigs with a coverage of below 0.5 (usually caused by inevitable contamination), (II) contigs with a coverage between 0.5× and 1.5× (chromosomal contigs, highlighted), (III) contigs with a coverage between 1.5× and 3× (chromosomal contigs, highlighted), (IV) contigs with a coverage between 3× and 50× (mtDNA), and (V) contigs with a coverage above 50× (ribosomal DNA).

In a third analysis, a contig-length vs. read-count plot was calculated based on an Illumina-only assembly. The plot illustrates the coverage of individual contigs compared to the average coverage. As we could show in previous studies for other fungi [23,37,38,45] most contigs are generally covered 0.5–1.5×. However, in diploid genomes, an additional group of contigs with coverage rates of 1.5–3.0× appears. This is also the case here, indicated as the groups II and III in Figure 1. Contigs of these two groups encode typical housekeeping genes and they almost represent the entire nuclear genome of *C. passeckerianus*. The two groups were interpreted as follows: group II comprises sequences of homologous chromosomes that are sufficiently different to be assembled as separate contigs, whereas group III consists of contigs with identical or nearly identical allelic variants of homologous chromosomes, which thus are covered by twice as many reads as group II contigs. In other words, the two haploid nuclei of *C. passeckerianus* are in parts so similar that they are represented on identical contigs (group III) in the dataset.

Taken together, the three analyses clearly demonstrate a diploid character of the sequence dataset. This reflects the dikaryotic state of the mycelium of *C. passeckerianus* and is in accordance to previous observations [4,5,6,7].

### 3.3. The Mitogenome Appears in Duplicate

One contig of the *C. passeckerianus* genome dataset was identified as the mitogenome, as described in the Material and Methods section. The GC content of the mitochondrial DNA (mtDNA) is only 27.1% and clearly differs from that of the nuclear genome (49.4%). Interestingly, this contig is unusually long (87 kb) and harbors two copies of the mitochondrial genes. Because such a duplication is very uncommon and could be an artifact of the assembly process, we manually bisected the contig to obtain two distinct circular mtDNA chromosomes (mtDNA-1 and mtDNA-2). They have the same coverage rate of sequence reads and slightly differ in size and in the sequence (Table 2 and Table 3). In both mtDNAs, 32 protein coding sequences and 26 sequences encoding tRNAs for all 20 amino acids were identified. Moreover, small and large rRNA loci are present in the mtDNAs. The small subunit ribosomal RNA genes (16 S rRNA gene = rns) are identical in both mtDNAs. Based on sequence comparisons with bacterial 16 S rRNA genes, the mitogenome of *C. passeckerianus* might originate from a bacterium related to ancestors of the family Peptoniphilaceae. The mitochondrial 16 S rRNA shows the highest degree of similarity (68%) to *Lagierella massiliensis* strain SIT14.

**Figure 2:**
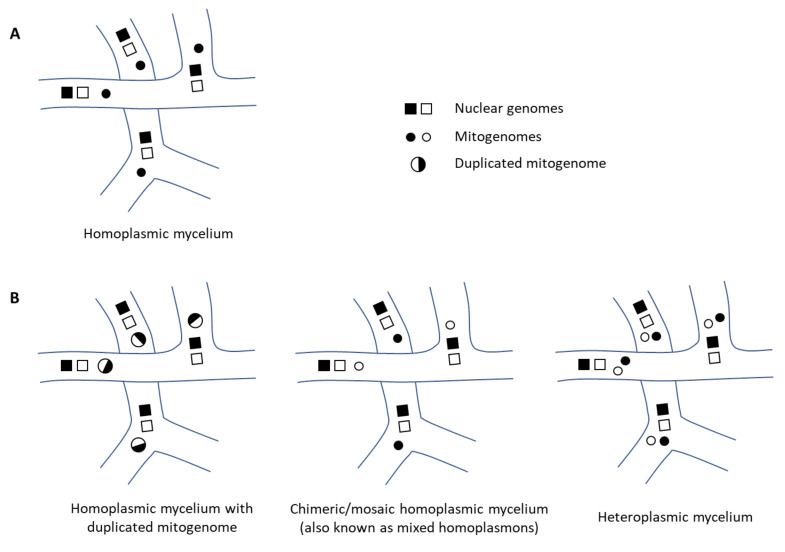
Distribution of mitogenomes and nuclear genomes in fungal mycelium. (**A**) Common scenario in Agaricales. (**B**) Possible scenario in *C. passeckerianus*.

The presence of two sets of mitochondrial genes is exceptional and leaves room for three interpretations (see Figure 2): (i) the mitochondrial chromosome consists of two consecutive repeats of mitochondrial genes making it twice as long as normal—as was the original outcome of the Nanopore sequence assembly, (ii) the mycelium is heteroplasmic containing two sets of different mitochondria per cell with ‘normal-sized’ chromosomes, or (iii) the mycelium is chimeric or mosaic with respect to the mitochondrion and comprises two sets of homoplasmic hyphae, each with a distinct ‘normal-sized’ mtDNA.

To ensure cellular integrity, mitochondria need to be inherited from the parental hyphae to the daughter hyphae. In Agaricales, the inheritance of mitochondria is best studied in laboratory mating experiments of *Agaricus* spp. [46,47,48], *Coprinus cinereus* [49,50], *Agarocybe aegerita* [51], and *Armillaria* spp. [8,9,10], from which the following general picture emerges. When two monokaryons with distinct nuclei and distinct mitochondria mate and fuse during anastomosis, the resulting hyphal compartment is dikaryotic and heteroplasmic [10,49]. However, this state is only transitional as the different mitochondria quickly segregate into subsequent hyphae that recover from the junction [46] and upon only a few events of cell division the proceeding dikaryotic mycelium is homoplasmic again [47,48], containing either one of the two mitochondria. In parallel, both of the nuclei usually spread reciprocally from the anastomosed compartment to the resident mycelium of the other mating type, respectively, while the mitochondria do not migrate [46,50]. As a consequence of both events—mitochondrial segregation in the novel hyphae and nuclear migration in the resident hyphae—the mycelium as a whole has become a chimeric or mosaic mycelium with respect to the mitochondria [9,49,50,51]. In contrast, the nuclear genome is identical within the—now completely dikaryotic—mycelium. Since both mating types can contribute their mitochondria to the homoplasmic progeny, this pattern of inheritance is probably best referred to as doubly uniparental [52]. Several experiments indicate that the chimeric state of the mycelium, however, is not permanent and one homoplasmon eventually outcompetes the other, which results in the dominant presence of one mtDNA [9,51,53]. This is in accordance with the observation that the vast majority of natural samples from Agaricales are predominantly homoplasmic [8,9,10,54,55]. With that in mind, and the fact that the DNA of *C. passeckerianus* had not been extracted from recently mated mycelium, the observed duplicated state of the mtDNA in *C. passeckerianus*—including all proposed interpretations—certainly displays a peculiarity and is a matter for further investigations.

### 3.4. Phylogeny and Comparative Genome Analysis Confirms Genetic Proximity of Clitopilus spp.

**Figure 3:**
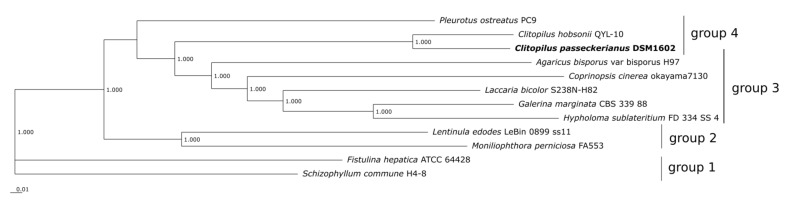
Phylogenetic tree of twelve selected Agaricales. The phylogenetic tree was build using SH-like local support values computed with ‘FASTtree 2.1′ within the comparative genomics tool ‘EDGAR 3.0′ on the bases of 1303 core genes. Branches show a perfect support value of 1.000.

In order to verify the taxonomic placement of *C. passeckerianus* within the basidiomycete order Agaricales, the comparative genomics platform EDGAR 3.0 [36] was applied. Eleven genomes of sequenced Agaricales members served as a comparison. The bioinformatic program calculated 1303 core genes for the selected species, on the basis of which a phylogenetic tree was computed. According to that, four groups can roughly be discriminated (Figure 3), at which *C. passeckerianus* clearly clusters with *C. hobsonii*, another representative of the species *Clitopilus* and the only one with an available genome sequence.

The other selected Agaricales are only distantly related to the genus *Clitopilus* and two fungi, *Fistulina hepatica* and *Schizophyllum commune*, are genetically so different that they appear as outgroups in the tree. The phylogenetic results are also reflected by calculations of genome-wide pairwise similarity, the Average Nucleotide Identity (ANI), the Average Amino Acid Identity (AAI), and the Percentage of Conserved Proteins (POCP) (see Tables S4–S6). These calculations disclose high values only for the closely related *Clitopilus* species. For example, *C. passeckerianus* shares 83.7% of its encoded proteins with *C. hobsonii* (based on POCP) and the ANI value of both genomes is 78.1%. As we could recently demonstrate, fungal strains or isolates from the same species have ANI values above 97% [22,38,56]. Likewise, POCP analysis were shown to be suitable to define genus boundaries. A threshold value of 70% is regarded appropriate to separate fungal genera from fungal species [21,22,38]. Accordingly, the investigations on genomic similarities clearly show that *C. passeckerianus* belongs to the same genus as *C. hobsonii* and both fungi constitute distinct species within this genus.

Similarities in the genomes of both *Clitopilus* strains were investigated further. To determine the conservation of gene blocks in the same relative position of both genomes, a macrosynteny analysis was carried out. This revealed a high degree of genomic synteny showing conserved chromosomes with only a few inversions, rearrangements of small areas, and some duplicated regions (see Figure S1). This makes a similar number of (non-homologous) chromosomes in both genomes likely, which in *C. hobsonii* has been indicated with 10 [20]. In order to compare functional genes of both strains, we needed to conduct a bioinformatic gene annotation also for *C. hobsonii*. In total, 12,973 genes were predicted. This is approx. half the number of genes predicted for *C. passeckerianus* (23,566, see above), which is due to the fact that *C. hobsonii* was sequenced as an artificial monokaryon instead of the natural dikaryon [20]. Based on the comparative genomics tool EDGAR [36], the two *Clitopilus* strains share 8654 core-genes, which represents approx. 75% of all *C. hobsonii* genes. These core-genes encode to a large extent basic cellular processes and play roles in general metabolism according to analyses with respective databases (such as COG, KEGG, and SWISS-PROT). However, both genomes also contain a noticeable number of genes with predicted functions in plant cell wall degradation, as judged by a dbCAN-CAZy [57] analysis (see Table S7). Besides the core-genes, *C. hobsonii* and *C. passeckerianus* harbor 4832 and 14,339 singletons (paralogous genes are not counted), respectively, and thus the pan-genome consists of 27,825 genes (4832 + 14,339 + 8654). Although some singletons could be assigned to KEGG pathways, most of them are annotated as hypothetical genes.

In summary, the comparative genome analysis illustrates that both species share a significant amount of genetic content. Simultaneously, a sufficient number of individual genes remain that potentially are associated with the different live styles of the fungi: in contrast to the saprotrophic *C. passeckerianus*, *C. hobsonii* has been observed to establish biotrophic symbiosis with plants [58].

### 3.5. Clitopilus passeckerianus Harbors a Huge Genetic Potential for Secondary Metabolites

**Figure 4:**
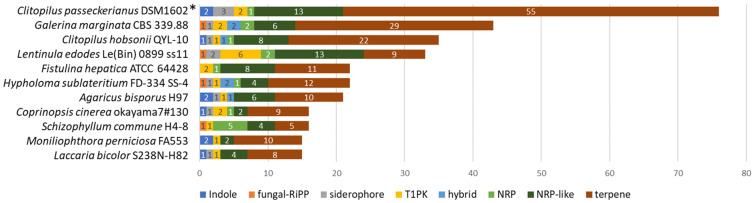
Distribution of BGCs in genomes of selected Agaricales. * Note that the genome of *C. passeckerianus* derives from the dikaryon and the actual number of BGCs presumably is even higher (see main text for details).

In order to detect biosynthetic gene clusters (BGCs), coding for terpenes, and other secondary metabolites, an analysis with the fungal version of the bioinformatics tool antiSMASH [59] was performed together with a BLAST search using the terpene synthase of the pleuromutilin BGC [17] from *C. pseudo-pinsitus* (accession number LC314149) as the query. This resulted in the large number of 76 BGCs present in the genome of *C. passeckerianus*, which displays a huge genetic capacity of the fungus to produce secondary metabolites. Especially outstanding is the high number of 55 terpene BGCs, of which two were assigned to the pleuromutilin biosynthesis (more details in the next chapter). In addition to *C. passeckerianus*, we investigated the distribution of BGCs in 10 further Agaricales genomes with the result that terpene BGCs generally were very abundant, particularly in *Galerina marginata* (29 clusters) and in *C. hobsonii* (22 clusters). However, none of them comprised a pleuromutilin BGC. Other frequently found BGCs were those coding for non-ribosomal peptides (NRP) and NRP-like metabolites, whereas BGCs for indoles, fungal-RiPPs, siderophores, type I polyketides, or hybrids thereof, were represented in the low single-digit level (Figure 4).

It needs to be considered that—in contrast to the other genomes investigated here, which are likely haploid monokaryons, as judged from their genomic size—the genome of *C. passeckerianus* derives from the dikaryon. Since the two haploid nuclei of the dikaryon are in parts so similar that they are represented on identical contigs in the dataset (see Section 3.2), the BGCs located on these contigs were identified only once by antiSMASH, although they would appear twice in the cell. Therefore, the total number of BGCs present in the dikaryotic genome of *C. passeckerianus* certainly is significantly higher than 76 and—consequently—the number of unique, non-homologous BGCs certainly is higher than half that value.

**Figure 5:**
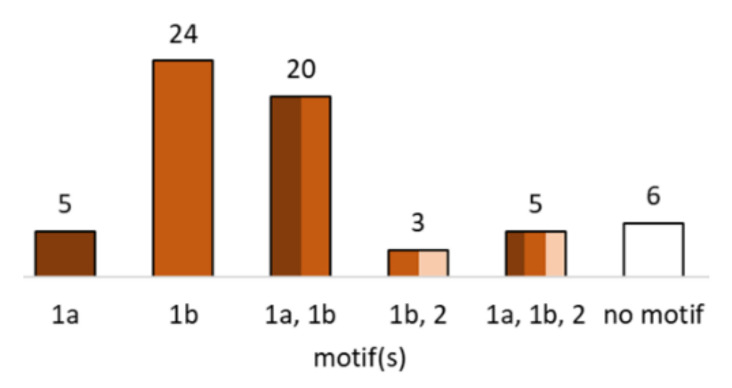
Number of predicted terpene synthases in *C. passeckerianus* with conserved motifs. 1a: class I motif DDXX(D/E); 1b: class I motif (N/D)DXX(S/T)XXXE; 2: class II motif DXDD.

The terpene BGCs in *C. passeckerianus* contain a total of 63 predicted terpene synthase genes. Terpene synthases catalyze dephosphorylation and coupling/cyclization reactions of linear isoprenoids of varying length such as GGPP. Two distinct classes of terpene synthases are distinguished by their substrate activation mechanism, which is either ionization-dependent (class I) or protonation-dependent (class II) [60,61]. In addition, bifunctional enzymes exist. Conserved motifs in the enzymes give hints to the reaction mechanism. The predicted terpene synthases of *C. passeckerianus* were screened for these motifs (see Figure 5). According to that, all but six contain at least one of the characteristic motifs and thus likely display functional enzymes. Moreover, the majority belongs to class I terpene synthases.

Terpenoid production is widespread in fungi [62,63]. Basidiomycetes in particular are known prolific producers [64] and an increasing number of basidiomycete terpene synthases are characterized [64,65]. Nevertheless, our swift analysis suggests that the actual potential to produce terpenoids by members of the Agaricales could even be higher than previously thought. In particular, the numerous terpene synthases in *C. passeckerianus* indicate a pronounced and versatile terpenoid metabolism. Considering that even very similar enzymes are able to produce a manifold terpenoid chemistry [60], it is very likely that terpenoids other than pleuromutilin will be isolated from this fungus in prospective studies.

### 3.6. Two Pleuromutilin Biosynthesis Gene Clusters Present in C. passeckerianus

**Figure 6:**
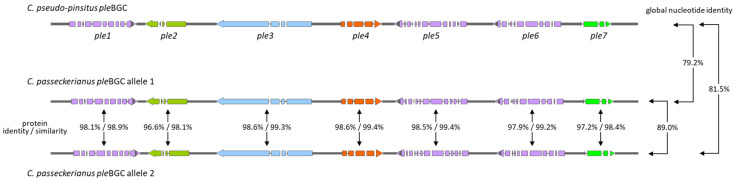
Pleuromutilin biosynthetic gene clusters (*ple*BGCs). Introns are illustrated as interruptions in the genes. Nucleotide identities and protein similarities/identities are indicated.

**Figure 7:**
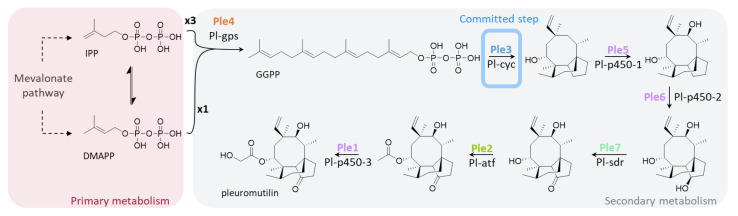
Pleuromutilin biosynthesis according to [16,17]. IPP: isopentenyl pyrophosphate; DMAPP: dimethylallyl diphosphate.

Two of the terpene BGCs were identified as pleuromutilin BGCs based on their similarity to the pleuromutilin BGC (*ple*BGC) of *C. pseudo-pinsitus* (Figure 6). Cluster architectures coincide and global nucleotide sequence identities are very high. The two *ple*BGCs of *C. passeckerianus* are localized on different contigs with a high degree of sequence identity and gene synteny, which is why they very likely represent allelic variants on homologous chromosomes of the dikaryotic fungus. Introns of the *ple* genes were verified by PCR amplification, cloning, and sequencing of *ple* transcripts, revealing intron numbers and positions that are in accordance with the findings of other groups [4,17] (see Figure 6 and Table 4). The function of *ple* genes in *C. passeckerianus* (Figure 7) has been proofed by Alberti et al. via heterologous expression of one of the clusters in *A. oryzae* [16]. Amino acid sequences of the *ple* gene products from both alleles are nearly identical, suggesting that either allelic cluster is able to autonomously give rise to pleuromutilin biosynthesis. This is in accordance with the fact that pleuromutilin is detected from monokaryotic cultures of *C. passeckerianus* [7].

### 3.7. Refactoring the pleBGC for Heterolgous Expression in Saccharomyces cerevisiae

Motivated by the success of heterologous pleuromutilin production in the Ascomycete host *Aspergillus oryzae* [16,17] and the recent advances of heterologous expression of basidiomycete terpene biosynthesis genes in yeast [66,67], we envisaged to reconstitute the *ple*BGC for heterologous expression in the fast-growing, well-studied and biotechnologically relevant yeast *Saccharomyces cerevisiae*.

**Figure 8:**
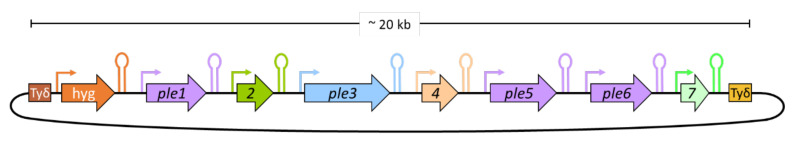
Schematic representation of a yeast-*ple*BGC. Tyδ: delta elements of the yeast retrotransposon Ty1; large arrows: genes; small arrows: yeast-specific promoters; hairpin structures: yeast-specific, high-capacity terminators. Expression cassettes are composed of a promoter, gen, and a terminator.

For this purpose, three composite pleuromutilin gene clusters consisting of various yeast-specific *ple* expression cassettes were conceptualized. The clusters were referred to as yeast-*ple*BGCs-A, -B, and -C, and contained all seven *ple* genes controlled by a different set of promoters, respectively (see Figure 8 and Table 5). Every *ple* gene should be flanked by its own promoter and terminator to avoid homologous sequences in the yeast-*ple*BGCs that may give rise to undesired recombination events in the final yeast host. Promoter sequences were taken from Maury et al., who identified *S. cerevisiae* promoters that were active at late stages during fermentation [68]. Usage of such promoters would allow the decoupling of cell growth from product formation, which is advantageous in industrial fermentation processes as the yeast metabolism is automatically channeled into the desired direction—i.e., biomass or pleuromutilin—during the individual fermentation steps. Given the anticipated functionality of both alleles of the *ple*BGCs in *C. passeckerianus*, one of the clusters was arbitrarily chosen as a basis for the yeast-specific *ple* expression cassettes. Furthermore, a set of seven high-capacity terminators was selected. Sequences of these terminators were taken from Curran et al., who identified terminators that increased mRNA half-life and improved gene expression in *S. cerevisiae* [69]. Besides the *ple* expression cassettes, the conceptualized yeast-*ple*BGCs comprised the hygromycin resistance cassette *hph*MX6 for future selection of transformants. Moreover, the clusters were flanked by delta elements of the yeast retrotransposon Ty1 in order to allow for stable (and multi-copy) chromosomal integration [70] of the yeast-*ple*BGCs in prototrophic *S. cerevisiae* wild-type strains.

In order to construct the designed yeast-*ple*BGCs, all the individual elements were PCR-amplified. In the case of the *ple* genes, the cDNA of *C. passeckerianus* served as a template to avoid the missplicing of the intron-dense *ple* genes. All other DNA elements were amplified from synthesized DNA molecules. In a next step, the discrete DNA elements (up to 22 per yeast-*ple*BGC) were step-wise assembled using a combination of fusion-PCR, type IIS restriction enzymes for seamless cleavage of intermediate products, and two consecutive rounds of Gibson assembly (see Figure S2 for more details). The final vectors carrying correctly assembled yeast-*ple*BGCs were identified by restriction digestion and the final sequence was verified by Sanger-sequencing of the entire composite gene clusters.

In summary, the pleuromutilin biosynthesis genes were successfully reassembled to yeast-specific gene clusters that are now ready for usage in future experiments. The step-wise Gibson assembly in combination with type IIS restriction enzyme digestion has proved to be a strong tool to put together 22 discrete DNAs to one large molecule. A high-quality genome sequence that includes information on the pleuromutilin biosynthesis genes—which we provided in the course of this study—has been the essential basis for that approach. In prospective studies, we will transfer the refactored yeast-*ple*BGCs into appropriate *S. cerevisiae* strains and evaluate heterologous pleuromutilin production.

## 4. Conclusions

In this study, a high-quality annotated genome of the pleuromutilin-producing fungus *C. passeckerianus* was established, providing a valuable resource for future transcriptomic, proteomic, and comparative genomic studies, which will help to understand the fundamental biological traits of this important pharmaceutical species. A multi-locus phylogenetic tree was prepared and enabled precise ranking within other sequenced genomes of Agaricales. The findings on duplicated mitochondrial genomes in *C. passeckerianus* sheds new lights on mitochondrial heritage in basidiomycetes. It remains to be seen whether similar mitochondrial features are going to be discovered in further dikaryotic genomes. Possibly, the heteroplasmic state is more widespread in basidiomycete fungi than previously assumed.

In addition, our study paves the way for studies aimed at interfering with the cellular metabolism to boost the production of pleuromutilin. Given the fact that protocols for genetic manipulation have been successfully developed, rational metabolic engineering approaches are now possible. In particular, the abundant terpene synthase genes are attractive targets as they display potential competitors for common precursors in the terpenoid metabolism. Therefore, silencing or deletion of (non-essential) terpene synthase genes should increase the substrate pool for pleuromutilin biosynthesis. Transcription analysis will help to identify the most competing pathways. Moreover, pathway genes of the primary metabolism such as the mevalonate pathway are now accessible and allow for a targeted manipulation of metabolic fluxes towards terpenoid biosynthesis [71,72].

Furthermore, in light of the plethora of BGCs that have been detected, our study revealed an extremely pronounced secondary metabolism of *C. passeckerianus*. Particularly, the large number of terpene synthase genes present in *C. passeckerianus* and related species suggests that much chemical and enzymatic diversity regarding terpenoids and their biosynthesis remains to be discovered. With respect to the fact that pleuromutilin is the only secondary metabolite known from *C. passeckerianus*, the majority of BGCs appear to be silent or only weakly expressed. Therefore, it seems obvious to assume that many more secondary metabolites await discovery using measures to activate these clusters (e.g., alternative cultivation conditions) or by applying sensitive analytical screening methods.

## Figures and Tables

**Table 1:** Sequencing and assembly statistics of the *C. passeckerianus* genome.

Feature	Value
Total number of raw bases (ONT)	22.81 Gb
Number of reads (ONT)	4.59 million
Total number of raw bases (Illumina)	4.70 Gb
Number of reads (Illumina)	15.7 million
Number of contigs	167
Largest contig	3,100,797 bp
Total length	65,616,532 bp
GC content	49.43%
N50 (shortest contig length at ½ genome length) ^1^	673,524 bp
Number of predicted genes	23,566

^1^ N50 can be described as a weighted median statistic such that 50% of the entire assembly is contained in contigs equal to or larger than this value [44].

**Table 2:** Features of the two mtDNA chromosomes.

Feature	MtDNA-1	MtDNA-2
Length	40.291 bp	40.382 bp
Number of CDS	32	32
Number of encoded tRNA	26	26
Number of encoded rRNA clusters	3	3
GC content	27.1%	27.1%

**Table 3:** Similarity of the two mtDNA chromosomes.

**Sequence Identity**	99.63%
**Alignment Length**	40,405 bp
**Identical Positions**	40,259 bp
**Gaps**	137

**Table 4:** The pleuromutilin biosynthesis genes in *C. passeckerianus*.

Gene Name	Alternative Name	Function	*ple*BGC	Length (bp)	Number of Introns	Protein Length (aa)	Nucleotide Identity	Amino Acid Identity (Similarity)
*ple1*	Pl-p450-3	Cytochrome P450	1	2107	10	523	93.9%	98.1% (98.9%)
			2	2108	10	522		
*ple2*	Pl-atf	Acetyltransferase	1	1301	3	377	93.7%	96.6% (98.1%)
			2	1304	3	377		
*ple3*	Pl-cyc	Terpene synthase	1	3041	3	959	96.7%	98.6% (99.3%)
			2	3040	3	959		
*ple4*	Pl-gps	GGPP synthase	1	1291	4	350	95.0%	98.6% (99.4%)
			2	1290	4	350		
*ple5*	Pl-p450-1	Cytochrome P450	1	2286	13	523	96.3%	98.5% (99.4%)
			2	2284	13	523		
*ple6*	Pl-p450-2	Cytochrome P450	1	2166	11	525	90.4%	97.9% (99.2%)
			2	2166	11	525		
*ple7*	Pl-sdr	Dehydrogenase	1	945	3	253	89.7%	97.2% (98.4%)
			2	945	3	253		

**Table 5:** Composition of the yeast-specific *ple* expression cassettes.

Promoters ^1^			Genes ^2^		Terminators ^3^
Yeast-*ple*BGC-A	Yeast-*ple*BGC-B	Yeast-*ple*BGC-C	All Yeast-*ple*BGCs		All Yeast-*ple*BGCs
pSPG4	pYIG1	pPGK1	*ple1*	(P450-3)	tCYC*
pCTA1	pYEF3	pTEF1	*ple2*	(acetyltransferase)	tVPS13
pACS1	pTDH3	pADH2	*ple3*	(terpene synthase)	tCPS1
pICL1	pADH1	pADY2	*ple4*	(GGPP synthase)	tPRM9
pADH2	pPGK1	pFBA1	*ple5*	(P450-1)	tHIS5
pATO1	pTEF1	pATO1	*ple6*	(P450-2)	tSPG5
pADY2	pFBA1	pCTA1	*ple7*	(dehydrogenase)	tUBX6

^1^ According to [68]. ^2^ Amplified from cDNA of *C. passeckerianus ple*BGC-1. ^3^ According to [69].

## Data Availability

The obtained genome sequences were deposited in the DDBJ/EMBL/GenBank database under the bioproject number PRJEB53944.

## Supplementary Materials

The following supporting information can be downloaded at: https://www.mdpi.com/article/10.3390/jof8080862/s1, Figures S1–S3: CPassGenomeSupplFigs.docx; Tables S1–S7: CPassGenomeSupplTabs.xlsx; Genbank File S1: yeast-pleBGC-A.gb; Genbank File S2: yeast-pleBGC-B.gb; Genbank File S3: yeast-pleBGC-C.gb.
