# Supplementary material for: Genome Assembly and Genetic Traits of the Pleuromutilin-Producer Clitopilus passeckerianus DSM1602
Source: J Fungi (Basel). 2022 Aug 16;8(8):862. doi: 10.3390/jof8080862 (PMC9410065; doi:10.3390/jof8080862)
Supplement: Supplementary file 1 [file jof-08-00862-s001.zip › 220705 CPassGenomeSupplFigs.pdf]

Supplementary Figures to:

Genome assembly and genetic traits of the pleuromutilin-producer *Clitopilus passeckerianus* DSM1602

Thomas Schafhauser, Daniel Wibberg, Antonia Binder, Christian Rückert, Tobias Busche, Wolfgang Wohlleben and Jörn Kalinowski

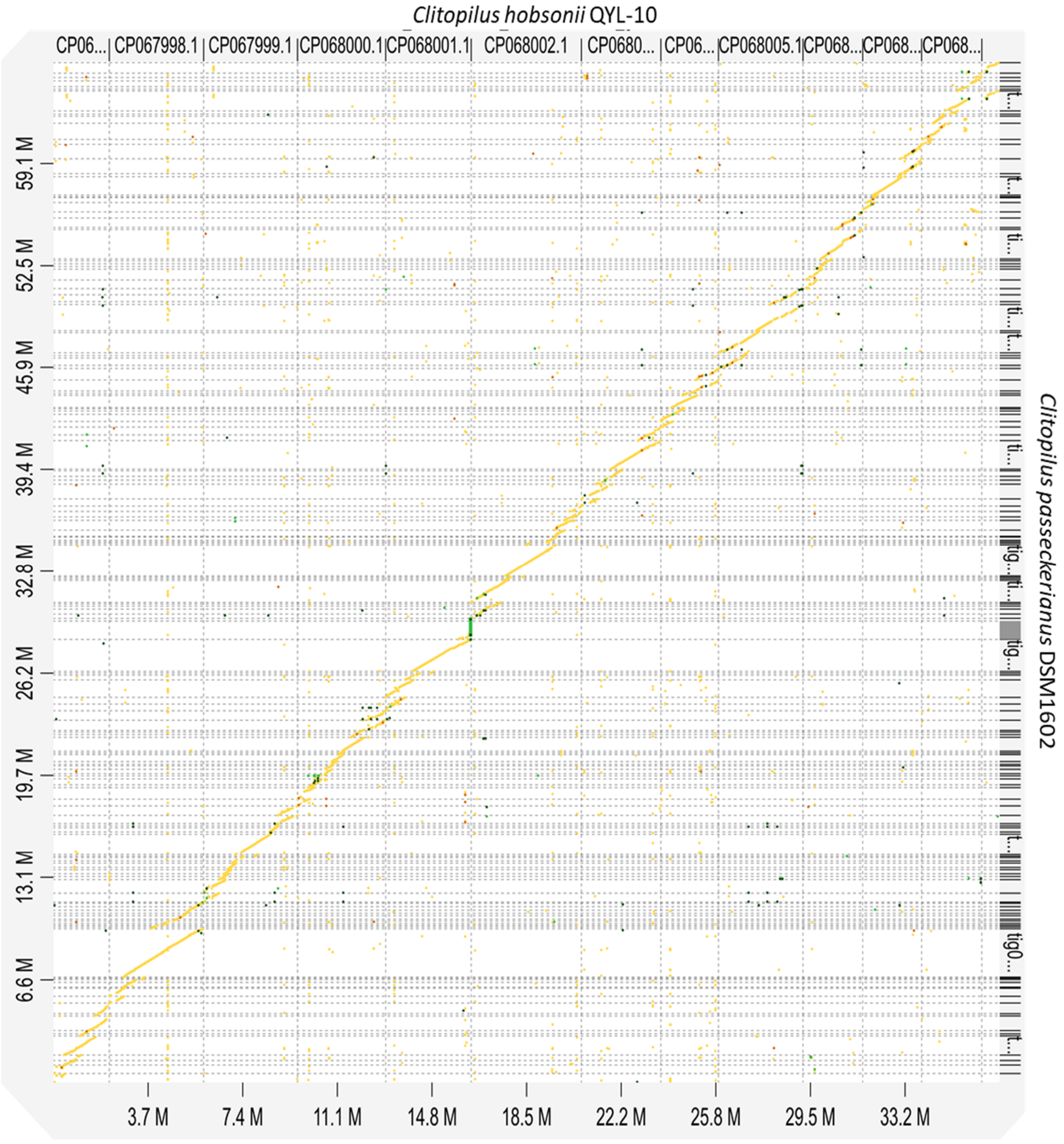

Figure S1. Macrosynteny plot between *C. passeckerianus* and *C. hobsonii*. Homologues sequence segments are plotted as small points according to their positions in the chromosomes of *C. hobsonii* (x-axis) and in the contigs of *C. passeckerianus* (y-axis). Larger homologous regions are displayed as yellow diagonal lines illustrating collinearity (= synteny) of the two sets of sequences. Accordingly, large parts of the contigs of *C. passeckerianus* are syntenic to chromosomes of *C. hobsonii*. Only very few regions are reversed complementary (displayed in green). Many parts of the *C. hobsonii* chromosomes are syntenic to

two regions of the *C. passeckerianus* sequence (this appears as parallel diagonal lines in the plot), which results from plotting a haploid against a diploid data set.

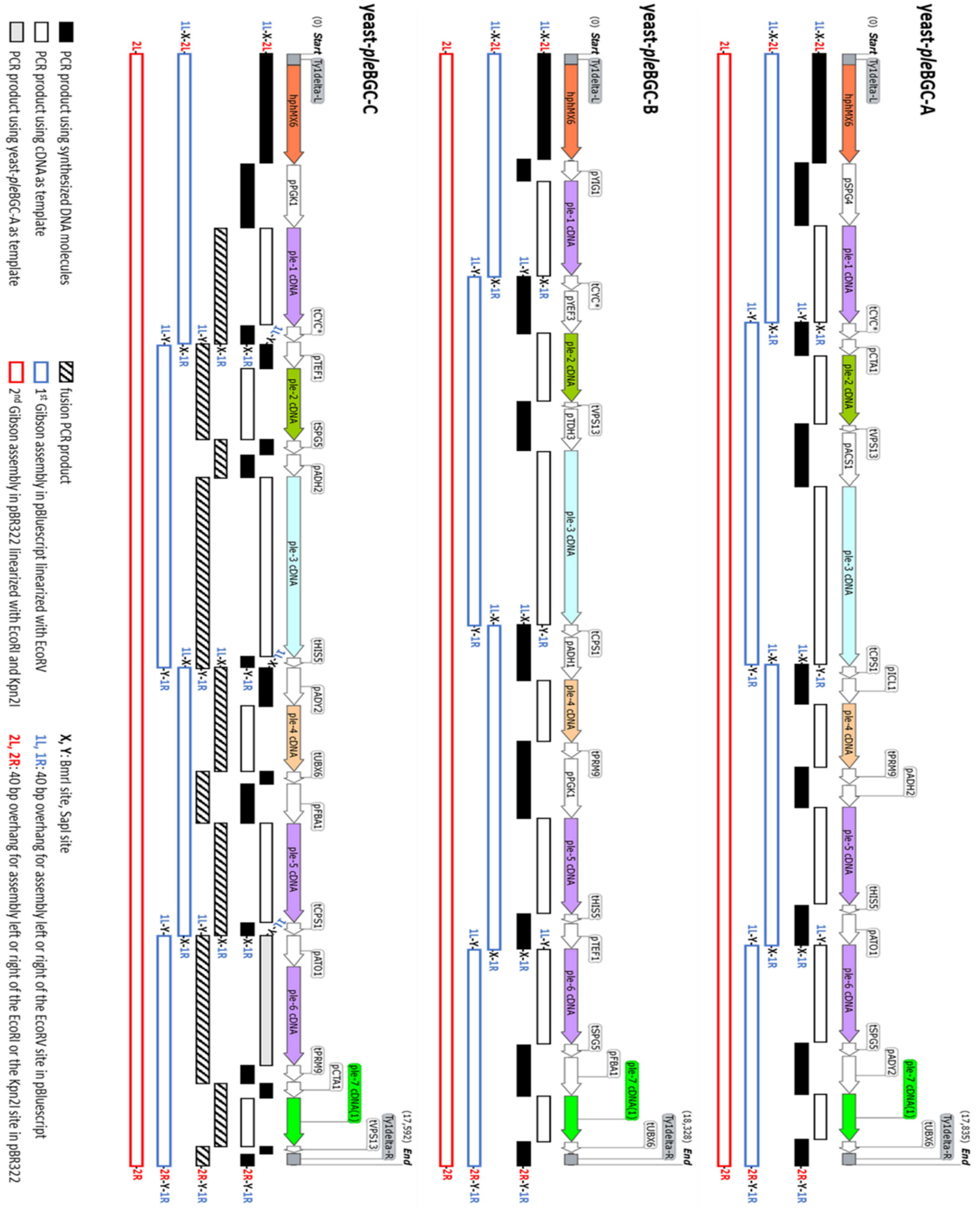

Figure S2. Step-wise assembly of the composite pleuromutilin gene clusters for heterologous expression in yeast. The architecture of the final clusters, yeast-*ple*BGC-A, -B, and -C, is shown in the upper line, respectively. PCR products are illustrated in white, black, or grey boxes underneath. Fusion PCR products are drawn as crosshatched boxes. The result of the 1st and 2nd Gibson assemblies are shown without plasmid backbone in blue and red, respectively. All PCR products contained extensions at their ends to allow restriction digestion, fusion or assembly. For fusion PCR and Gibson assembly, the individual DNA molecules overlapped in a length of 40 bp. Sequences that are homologous to parts of the

cloning vectors pBluescript and pBR322 are designated 1L (5′-CGGGCCCCCCCTCGAGGTCGACGGTATCGATAAGCTTGAT-3′), 1R (5′-ATCGAATTCCTGCAGCCCGGGGGATCCACTAGTTCTAGAG-3′), 2L (5′-AAAATAGGCGTATCACGAGGCCCTTTCGTCTTCAAGAATT-3′), and 2R (5′-CCGGATCTGCATCGCAGGATGCTGCTGGCTACCCTGTGGA). Restriction sites for the type IIS endonucleases BmrI (5′- ACTGGGNNNNN-3′) and SapI (5′-GCTCTTCNNNN-3′) are draw as ‘X’ or ‘Y’.

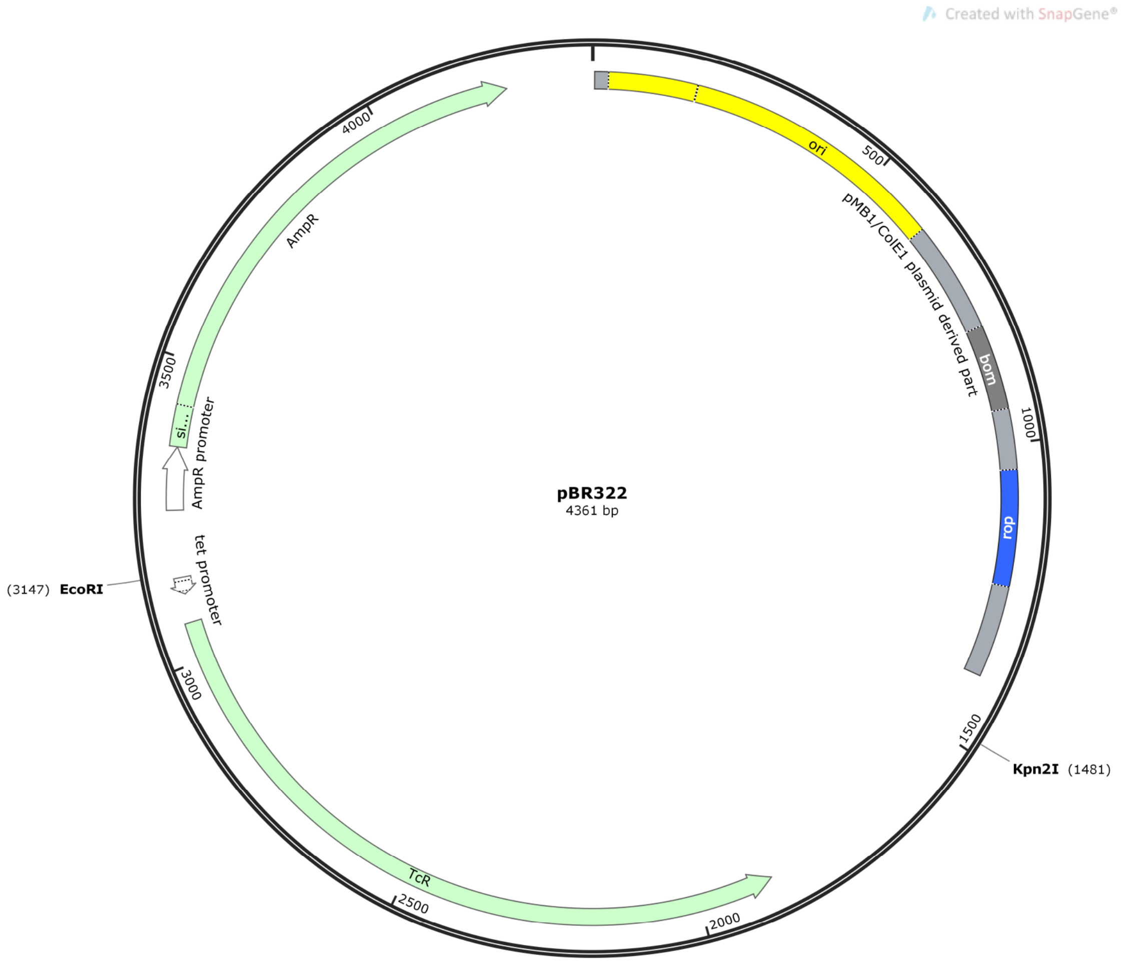

Figure S3. Map of the vector pBR322. AmpR: beta-lactamase gene; ori: high-copy-number ColE1/pMB1/pBR322/pUC origin of replication; bom: basis of mobility (relict from original plasmid), rop: gene coding for 'repression of priming'-protein that reduces plasmid copy number (--> ORF towards bom); TcR: gene coding for tetracycline efflux protein.
